# UnetDVH-Linear: Linear Feature Segmentation by Dilated Convolution with Vertical and Horizontal Kernels

**DOI:** 10.3390/s20205759

**Published:** 2020-10-11

**Authors:** Jiacai Liao, Libo Cao, Wei Li, Xiaole Luo, Xiexing Feng

**Affiliations:** State Key Laboratory of Advanced Design and Manufacturing for Vehicle Body, Hunan University, Changsha 410006, China; ljc_hnu@hnu.edu.cn (J.L.); lw_hnu@hnu.edu.cn (W.L.); luoxiaole@hnu.edu.cn (X.L.); jeremyfeng@hnu.edu.cn (X.F.)

**Keywords:** neutral networks, semantic segmentation, dilated convolution, linear features

## Abstract

Linear feature extraction is crucial for special objects in semantic segmentation networks, such as slot marking and lanes. The objects with linear characteristics have global contextual information dependency. It is very difficult to capture the complete information of these objects in semantic segmentation tasks. To improve the linear feature extraction ability of the semantic segmentation network, we propose introducing the dilated convolution with vertical and horizontal kernels (DVH) into the task of feature extraction in semantic segmentation networks. Meanwhile, we figure out the outcome if we put the different vertical and horizontal kernels on different places in the semantic segmentation networks. Our networks are trained on the basis of the SS dataset, the TuSimple lane dataset and the Massachusetts Roads dataset. These datasets consist of slot marking, lanes, and road images. The research results show that our method improves the accuracy of the slot marking segmentation of the SS dataset by 2%. Compared with other state-of-the-art methods, our UnetDVH-Linear (v1) obtains better accuracy on the TuSimple Benchmark Lane Detection Challenge with a value of 97.53%. To prove the generalization of our models, road segmentation experiments were performed on aerial images. Without data argumentation, the segmentation accuracy of our model on the Massachusetts roads dataset is 95.3%. Moreover, our models perform better than other models when training with the same loss function and experimental settings. The experiment result shows that the dilated convolution with vertical and horizontal kernels will enhance the neural network on linear feature extraction.

## 1. Introduction

Linear features are essential in practical applications, such as parking slot detection [1,2], lane segmentation [3,4,5] and road segmentation in aerial images [6,7,8,9]. The objects with strong edge features can use some conventional operators to describe them. Researchers have extensively explored linear feature extraction methods based on traditional algorithms and deep learning models. The method proposed in this article is related to these methods.

Traditional methods encourage researchers to propose various morphological operators to extract target edges with linear features. Edge detectors are commonly used to preprocess the image for linear feature extraction, such as Canny [10] and Sobel [11]. The morphological operators can use the structuring element to extract image regions with a similar shape, and thus grey-level hit-or-miss transform [12,13] and top-hat [14] transform can utilize the linear structuring element to extract linear features, but it is difficult to determine the perfect value for thresholding. Both edge detectors [10,11] and morphological operators [12,13,14] have the advantage of a larger receiving field in the horizontal and vertical directions. We utilized and enhanced this advantage to design a new convolution block. More advanced feature extractors like HOG and Haar features [15], can not only extract linear structure features but also combine edge information with pixel information of local regions. These advanced feature extractors have the problem of being unable to learn high-dimensional features. The designed convolution block will perform the same operations as these advanced feature extractors, but it can learn high-dimensional feature information. Multiple different feature operators can also be used in combination to describe the symmetry and posture information of objects. A more direct way to detect straight lines is to use Hough transforms [16], and Radon-and-Hough-transform [17,18]-based algorithms were proposed to detect straight lines, circles, and ellipses. However, these spatial transformation methods require us to input hyperparameters such as the length and the interval of the detected line. The designed convolution block only needs to set the parameters of kernels, which are simpler than the methods based on Hough transforms [16,17,18]. The traditional linear feature detection method is based on our priori knowledge and observation results, which is simple and effective for special applications.

With the widespread usage of deep learning [19], we gradually ignored the role of a priori feature, because deep neural networks can learn far more feature information than the artificially designed feature filters. A deep convolutional neural network is composed of thousands of convolution kernels, and each of them performs as a feature descriptor, and the convolutions can learn appropriate feature representations for the problem end-to-end instead of using hand-crafted features that require domain expertise. A 3×3 convolution kernel is at the same size as the traditional feature extractor of the Sobel filter. Sobel [11] filters are similar to manually designed deep convolution kernels, which specify the internal values of its kernels. Like other deep learning methods, the designed convolution block will automatically learn the internal parameters of the convolution kernels, which is more flexible than the Sobel filter. Convolution kernels with functions like pooling [20], dilated convolution [21], and upsampling have appeared. Different convolutions in the neural network learn the parameters through training methods. It is the biggest difference from the feature extraction filter designed by customary methods.

There are two main problems in this research. First, many traditional methods based on geometric shapes [10,11] and structuring elements [12,13,15] are utilized to extract linear features, but they are relatively inefficient. Second, linear features require a larger receptive field, but the current networks do not give full consideration to this.

The main features of our model are that the high-fusion layers behind the Unet [22] can fuse multi-scale upsampling and skip connection channel features. We add a dilated convolution block with horizontal and vertical kernels before the upsampling layer, which can not only increase the receptive field but also enhance the extraction of linear features. We propose a method of mixing the DVH and the v9h9 for linear feature extraction. Putting the DVH into the encoder layer can minimize the damage to the information learned from the lower layers, and the v9h9 can only be placed behind the encoder layers.

The content and expected contributions of this research are listed as follows:

- A new method is proposed based on spatial convolution kernels for linear feature segmentation. It utilizes the dilated convolution [21] with a vertical and horizontal convolution block (DVH) in semantic segmentation networks. This method is more efficient than the traditional vertical and horizontal convolution kernels. Traditional vertical and horizontal convolution that use the size of 9 × 1 and 1 × 9 (v9h9) kernels can be replaced by the proposed dilated convolutions with vertical and horizontal convolution (DVH) kernels of the size 3×1 and 1×3. The latter becomes more stable and is able to obtain better results on different datasets. In addition, the DVH block can be inserted into other backbone semantic segmentation networks to improve the linear feature segmentation capabilities of the segmentation networks;
- We have designed a series of experiments to observe how the positions of the DVH and v9h9 blocks impact the semantic segmentation networks for linear feature extraction. Adding spatial convolution kernels to neural networks for feature extraction is of great significance for future research.

This paper is organized as follows: Section 2 gives an overview of the research works related to linear feature extraction. Section 3 introduces the details of the proposed method, including VH-stage, different types of horizontal and vertical convolutions, and training loss functions. In Section 4, we evaluate our models on different public datasets and discuss the experimental results. We visualize the feature map and explain how the proposed DVH module plays a role in the linear feature extraction process. Section 5 presents the conclusions of our study.

## 2. Related Works

Deep networks based on the Fully Convolutional Network (FCN) [23,24] have become the defacto of image semantic segmentation in recent years. The semantic segmentation network classifies each pixel in the image and shares a calculation method with the traditional feature extraction operators, which convolves the pixel with the surrounding areas. Many new models are applied to accomplish the segmentation task based on this idea. A traditional deep semantic segmentation network consists of two modules, downsampling and upsampling, such as the encoder–decoder framework. The input image will go through an encoder first, then connect to the decoder through a central node. U-net [22] proposed combining the high-resolution features of the encoder with the outputs of the decoder. In addition to VGG [25], other efficient classification models can be used to implement the feature extraction in the encoding module, such as ResNet [26] and DenseNet [27]. However, these backbone feature extraction networks mostly stack local convolutional operations, and thus are hardly able to well cope with complex scenes with a variety of different categories due to the limited effective fields-of-view. Dilated convolutions with larger dilation rates have wider receptive fields without additional cost or overly downsampling the feature maps [21,28,29]. Dilated convolutions and multi-scale context aggregations [30,31,32] are also tightly coupled. However, dilated convolution has limitations. The input feature map is in a square window, and this will increase the receptive field while absorbing some irrelevant information from irrelevant regions [32,33] for some features with long-distance edges (lanes, slot markers, etc.). The proposed method makes use of dilated convolutions with a strip shape.

Concerning linear features, the convolution networks need a larger receptive field. The kernel sizes we often use in convolutions are 3×3, 5×5, and 9×9. To enhance the linear feature-learning ability of convolutions in the horizontal and vertical directions, VH-HFCN [2] proposed using 1 × 9 and 9 × 1 kernels (v9h9) in traditional convolutions, while SPnet [34] uses 1 × 3 and 3 × 1 convolution kernel in the pooling layers. The designed block has a structure similar to VH-HFCN [2] and SPnet [34]. It is demonstrated that vertical convolution kernels with the size of 9×1 and horizontal convolution kernels with the size of 1×9 between encoder and decoder will improve the parking slot marking segmentation performance [2]. Our proposed method designs a block similar to the v9h9 block for linear feature extraction. The v9h9 module obtains global context information by increasing the kernel size of traditional convolutions. The authors of [21] developed a dilated convolution module that aggregates multi-scale contextual information without losing resolution or analyzing rescale images. This is a more efficient method to support the exponential expansion of the receptive field without any loss of resolution. Guided by dilated convolution [21,35] and the VH-stage, we designed the DVH block to improve the semantic segmentation network for linear feature extraction. HFCN [36] comes up with a further structured layer based on FCCN [37], and each unpooling layer follows a combination layer. This method can fuse upsampling features of different receptive fields in high-fusion layers. In addition to increasing the receiving range of the feature extraction network, we also added high-fusion layers behind the Unet to fuse multi-scale upsampling features.

## 3. Proposed Method

### 3.1. An Overview of the Method

**Figure 1:**
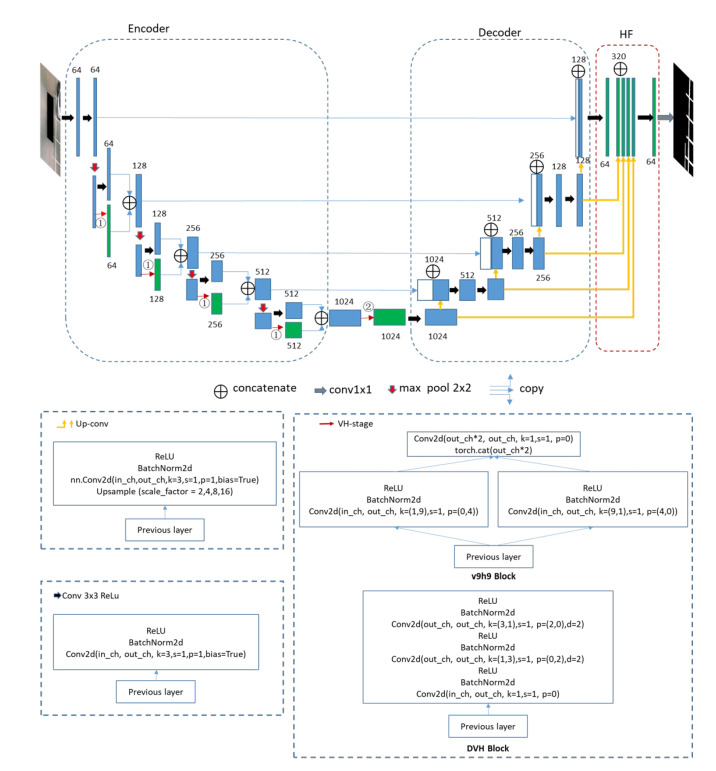
An overview of the proposed structure of segmentation network. An extra HF modile, which was proposed in [36], is added behind each upsampling layer of the Unet. In addition, we changed the Unet basic structure in ➀ and ➁, and putt our spatial horizontal and vertical convolution blocks at these places. The green rectangle represents the convolutional layers we added. The conv1x1 represents the convolution with a kernel size of 1 × 1. It will not change the input feature map size. There are two different blocks in the VH-stage: v9h9 block and DVH block. We only use one of them in the VH-stage; the v9h9 block contains the vertical and horizontal convolution with a kernel size of 9 × 1 and 1 × 9, respectively. The DVH block consists of the vertical and horizontal convolution with a kernel size of 3 × 1 and 1 × 3, respectively. The dilatation rate is 2. The Up-conv contains an upsample layer, and the scaling factor is a multiple of 2.

Our semantic segmentation network structure is shown in Figure 1. We marked two points in this traditional network structure diagram to indicate where we may want to change. In the research work, we selected three different datasets related to linear feature segmentation. The SS dataset [1] contains a lot of parking slot markers and the TuSimple lane dataset [38] consists of road lanes. The Massachusetts Roads dataset contains a large number of aerial images with marked roads [39]. These datasets contain typical segmentation targets with distinct linear features. The backbone network of Unet [22] is commonly used in different tasks of semantic segmentation networks. The research in [1] found that the pixels of the slot marking segment made significant location errors. That’s because the width of the slot marking is only six pixels, but the upsampling rate should be 8×. We utilized the Unet whose upsampling rate 16× to minimize the location error, and a highly fused convolutional network is added behind the Unet final upsampling layers. There are five upsampling blocks in the upsampling part that reserve feature information within different scales. We have the up-conv block proposed by Yang et al. [36], named HF layers, which can fuse multi-scale upsampling information. The proposed VH-stage in [1] solves the problem that short kernels are not enough to cover full linear features, while long kernels may reduce the efficiency of the network, even reducing the segmentation performance. The longer the convolution kernels, the better the segmentation results for linear feature segmentation. The block v9h9 means horizontal kernels are within a size of 1 × 9 and vertical kernels within a size of 9 × 1. VH-HFCN [2] put the v9h9 block behind the encoder layers. We fully explored how the VH-stage’s position will influence the semantic segmentation networks in this paper. We designed a more useful block to learn long-range information based on dilated convolution, and we call it the DVH block. The basic convolution kernel parameters and composition of all basic blocks used in our models are shown in Figure 1.

There are three differences between the proposed method and the VH-HFCN method: First, in the VH-Stage, VH-HFCN uses traditional convolution kernels with sizes of 9 × 1 and 1 × 9, while we use dilated convolutions; the kernel size is 1 × 3 and 3 × 1. Second, the model we designed also tried to use both horizontal and vertical convolution in and behind encoder layers. Third, VH-HFCN finally uses addition operations to fuse feature information from the encoder and the corresponding decoder layers. We kept the origin structure of Unet that concatenated the encoder feature maps and the corresponding decoder feature maps. In this way, we can retain the information learned from the low layers independently and do not damage the information learned from the lower layers.

### 3.2. VH-Stage

**Figure 2:**
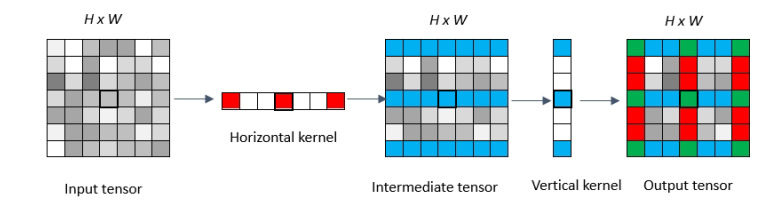
The vertical convolution is a dilated convolution with kernel size of (3,1), the dilated factor *l* is (2,0); the horizontal convolution is a dilated convolution with kernel size of (1,3), the dilated factor is (0,2). The blue grid represents the feature values activated by the vertical convolution kernel. The red grid represents the feature values activated by the horizontal convolution kernel, and the green grid represents the feature values activated by both horizontal and vertical convolution kernels.

An extra VH-stage is added behind the encoder layers to learn linear features in [1]. By adding this extra VH-stage, which is composed of special design kernels, the segmentation performance can be improved. The v9h9 block is designed for long-range information extraction, and its core idea is to increase the receptive fields of the convolution kernels. However, the experimental result has verified that the maximum limit kernel size for VH-stage is 9 [2]. Inspired by VH-stage and dilated convolutions, we create a DVH block for linear feature segmentation. As mentioned in the inception model [40], the 1×n and n×1 filters can make the model easier to train. Further, 1×n and n×1 filters in dilated convolutions will increase the receptive fields of backbone networks, and this will help the model to explore more complex features with more convolution layers. The receptive field of the network can be recursively calculated by the following formula

(1) $$\begin{array}{c} r_{n}=r_{n-1}+\left(k_{n}-1\right)\times d_{n}\times \prod_{i=1}^{n-1}s_{i} \end{array}$$

where $r_{n}$ represents the receptive field of the *n*th feature map. $d_{n}$ represents the dilated factor of the *n*th feature map. $s_{i}$ is the stride in *i*th convolution layer, and $k_{n}$ denotes the kernel size of *n*th convolution. The DVH block will use the dilated convolution with a kernel size of 1×3 to extract horizontal linear features. Similarly, dilated convolution kernels with a kernel size of 3×1 help extract vertical linear features. To keep the feature map size unchanged before going through max pooling layers, the vertical and horizontal dilated convolution kernels with padding size should be (2,0) and (0,2), respectively. The dilated rate is 2 for both vertical and horizontal convolution. Thus, the receptive field of our DVH block is 7×7. Figure 2 depicts our proposed DVH. Let $x\in R^{C\times H\times W}$ be an input tensor, where *C* denotes the number of channels. We first feed x into a horizontal convolution layer, then go into a vertical convolution layer. $f_{v}\left(x\right)\in R^{C\times H}$ is vertical convolution, $f_{h}\left(x\right)\in R^{C\times H}$ is horizontal convolution. An output $z\in R^{C\times H\times W}$ is computed as

(2) $$z=f_{v}(\sigma \left(\left(f_{h}\left(x\right)\right)\right)$$

where σ is the ReLU function. It should be noted that there is one pathway to combine the features extracted by vertical and horizontal convolution layers.

### 3.3. The Position of the DVH Block

A VH-stage with a v9h9 block is put behind the encoder layers in VH-HFCN [1]. This extra stage hardly destroys the feature information learned in encoder layers. Our proposed method tries to put the DVH block in and behind encoder layers. We add a branch in each encoder layer to avoid destroying feature information in lower layers, because the branch structure ensures that the learned information transmitted from the upper layer will independently pass through two different channels. One channel is a traditional convolution layer, and the other is a layer with horizontal and vertical convolution kernels. Besides this, horizontal and vertical convolution layers will not affect the feature extraction of another channel. Finally, the output feature maps from two independent channels will be concatenated together, and this ensures that the final output of each coding layer retains the convolution features of two separate channels. Therefore, the added branch will ensure that the information in the traditional convolutional layer will not be used to extract linear features within each coding layer.

- Putting the DVH block in the encode layers at ➀ is shown in Figure 1. The feature extraction and the outputs of the network we designed can be expressed by the following formula
  (3) $$\begin{array}{cc} \; & E\;(x)=E{}_{ncoder}\left[Cov\;(x),DVH\;(x)\right]\\ \; & D\;(x)=D{}_{ecoder}\left[Cov\;(x),DVH\;(x),E\;(x)\right]\\ \; & H\;(x)=H{}_{fusion}\left(D\;(x)\right)\\ \; & F_{out}=F_{net}\left[H\;(x),D\;(x)\right] \end{array}$$
  In the above equations, *x* is the input of our proposed networks, Cov(x) represents the traditional convolution block, while DVH(x) represents the DVH block. $E_{ncoder}$ and $D_{ecoder}$ represent the encoder and decoder layers in the backbone network. $H_{fusion}$ means the high-fusion layer behind the up-sampling operation. E(x),D(x) and H(x) are the output of the corresponding network layer. $F_{out}$ represents the final output, while $F_{net}$ represents the whole neural networks.
- Putting the DVH block after the encoder layer at ➁ as shown in Figure 1, the feature extraction and output of the network we designed can be expressed by the following formula
  (4) $$\begin{array}{cc} \; & E\;(x)=E{}_{ncoder}\left[Cov\;(x)\right]\\ \; & D\;(x)=D{}_{ecoder}\left[Cov\;(x),DVH[E\;(x)],E\;(x)\right]\\ \; & H\;(x)=H{}_{fusion}\left[D\;(x)\right]\\ \; & F_{out}=F_{net}\left[H\;(x),D\;(x)\right] \end{array}$$
- Putting the DVH block in and behind the encode layer at ➀ and ➁ is shown in Figure 1. The feature extraction process and output of the network can be expressed by the following formula
  (5) $$\begin{array}{cc} \; & E\;(x)=E{}_{ncoder}\left[Cov\;(x),DVH\;(x)\right]\\ \; & D\;(x)=D{}_{ecoder}\left[Cov\;(x),DVH[E\;(x)],E\;(x)\right]\\ \; & H\;(x)=H{}_{fusion}\left[D\;(x)\right]\\ \; & F_{out}=F_{net}\left[H\;(x),D\;(x)\right] \end{array}$$

We can conclude from Equations (3)–(5) that the location of the DVH block will affect the feature extraction and information exchange in the networks.

We replaced the DVH block with the v9h9 block and found out how the DVH block will influence the results of the final output. We also combined different positions of v9h9 and DVH to explore the effect of different convolution kernels at different positions on the final segmentation result, because the altered positions and blocks of VH-stage affect the feature extraction process of the entire network.

### 3.4. Loss Function

The inputs of our models are images and ground truths. We use the BCELoss [41] and Dice loss [42,43] as our loss function. The BCELoss is designed for binary classification. The BCELoss with sigmoid function can be defined as follows

(6) $$L_{bce}=-\frac{1}{N}\sum_{i=1}^{N}y_{i}\cdot \log\left(p\left(y_{i}\right)\right)+\left(1-y_{i}\right)\cdot \log\left(1-p\left(y_{i}\right)\right)$$

where $L_{bce}$ represents the binary cross-entropy loss function with a sigmoid activate layer, *y* is the label (1 for positive points and 0 for negative points); p(y) represents the predicted probability of the point, and it is positive for all *N* points. The dice loss is proposed to solve the problem that the training of the network will become stuck in local minima and will be biased towards the background, because almost 90% of the pixels in nature of the ground truth images are background, while the remaining pixels are foreground (vessels). The differentiable approximation of dice loss is defined as

(7) $$L_{dice}=1-\frac{2\sum_{x\in \Omega }p_{l}\left(x\right)g_{l}\left(x\right)}{\sum_{x\in \Omega }p_{l}^{2}\left(x\right)+\sum_{x\in \Omega }g_{l}^{2}\left(x\right)}$$

where pl(x) provides the probability of the pixel *x* that belongs to class *l*. gl(x) is a vector of the ground truth label, one for true class and zero for other classes. In this case, the loss function in our training process can be defined as

(8) $$L=L_{bce}+L_{dice}$$

### 3.5. Datasets and Experimental Settings

#### 3.5.1. Datasets

Our models currently have no real application, but can provide preprocessing results for other real application tasks. For example, the results of lane segmentation can be used for lane classification and 3D lane detection tasks [38,44], while the segmentation results of parking slots can be used for parking slot detection and automatic parking localization [1,45]. All our experiments are implemented on public datasets. We firstly experimented on datasets with typical linear features, such as lanes and parking slots. To further explore the segmentation performance of the model on geometric shapes, we conducted experiments on the road segmentation dataset. The public datasets we used are the following:

- The SS dataset [1]: The SS dataset is composed of Around View Monitor (AVM) images and corresponding annotation images that are collected from various parking conditions outdoors and indoors. This dataset contains 6763 camera images with 320×160 pixels. The number of training images and test images is 4057 and 2706, respectively, among which there are four categories: free space, marker, vehicle, and other objects. Each image has a corresponding ground truth image that is composed of four-color annotations to distinguish different classes. In particular, the indoor samples are difficult to discern because the reflected light seems similar to slot markers, hence degrading the detection of slot markers;
- The TuSimple lane dataset (http://benchmark.tusimple.ai/): This dataset released approximately 7000 one-second-long video clips of 20 frames each, and the last frame of each clip contains labeled lanes. This dataset contains complex weather, different daytimes, and different traffic conditions with 6408 1280 × 720 images, separated into 3626 for training, and 2782 for testing. The types of annotations are polylines for lane markings. All the annotations information is saved in a JSON file to guide researchers in how to use the data in the clips directory. The annotations and testing are focussed on the current and left/right lanes. There will be, at most, five lane markings in ‘lanes‘. The extra lane is used when changing lanes since it is confused to tell which lane is the current lane. The polylines from the recording car are organized by gaps at the same distance (’h_sample’ in each label data), and 410 images are extracted from the training set used as a validation set during training;
- The Massachusetts Roads Dataset [39] (https://www.cs.toronto.edu/~vmnih/data/): The Massachusetts Roads Dataset consists of 1171 aerial images. On the road data, each image is 1500 × 1500 pixels in size. The dataset is randomly split into a training set of 1108 images, a test set of 49 images, and a validation set of 14 images. The dataset covers a wide variety of urban, suburban, and rural regions with an area of over 2600 km${}^{2}$. With the test set alone covering over 110 k^2^, this is by far the largest and the most challenging aerial image labeling dataset.

**Figure 3:**
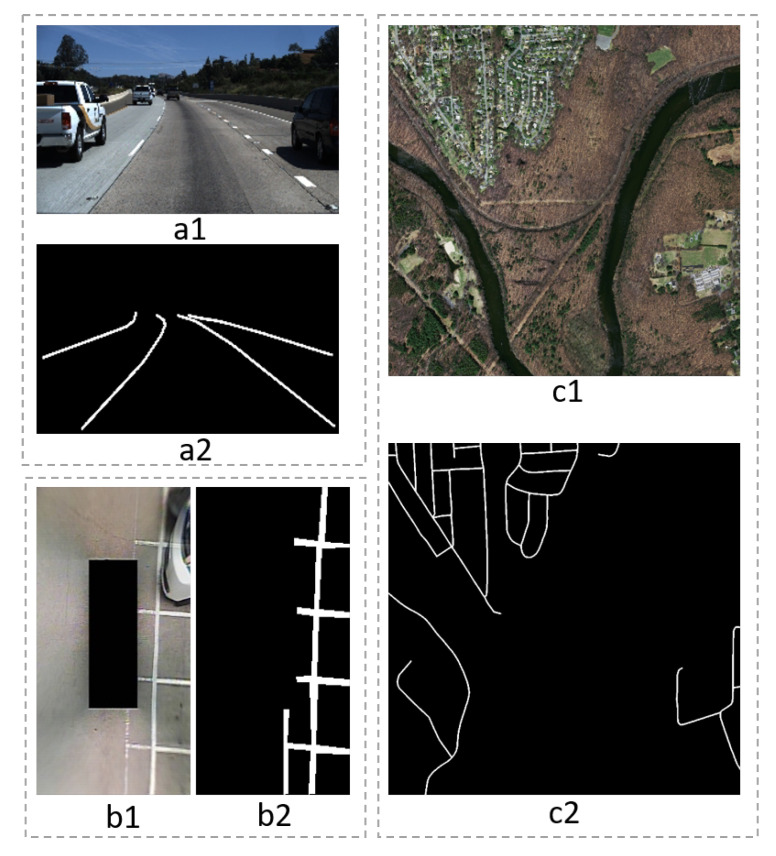
Example of the training dataset: a1 and a2 are the image and mask of TuSimple lane dataset, respectively; b1 is an image of the SS dataset, and b2 is a mask of b1 which only contain parking slot makers; c1 and c2 are the image and mask of the Massachusetts Roads Dataset, respectively.

The training and test set are allocated according to the division of the original dataset. Reference [1] provided detailed information on the division of the SS dataset. Similar to other research work [38,46], the original training set and test set for the TuSimple dataset remain unchanged. The width and height of the Unet input image must be an integer multiple of 16. We need to normalize images of different sizes before training. We processed the TuSimple data set, and the size of the input image is 256 × 160. The input size of the SS dataset is 320 × 160. The input size of the Massachusetts Roads Dataset is 640 × 640. Examples of images and masks in different datasets are shown in Figure 3.

The SS dataset contains different types of road marking, including parking slots, some basic steering signs, and zebra crossings as well. The TuSimple lane dataset contains various road lanes with dashed and solid lines. These two datasets contain straight lines with different categories, width, colors, and viewing angles. Unlike the first two datasets, the Massachusetts Roads Dataset collect aerial images for road segmentation. These three different datasets can evaluate our model more comprehensively under various scenes.

#### 3.5.2. Experimental Settings

We implemented our experiments based on Python code and Pytorch framework [47] in Ubuntu 16.04, and PyTorch is a deep learning framework written in Python language. The hardware configuration is as follows: NVIDIA RTX2080 graphics card, 10 GB GPU memory, i9-7900X @3.60GHz×10 processors, and 32 GB RAM.

Data augmentation is a common technique that has been proven beneficial for the training of machine learning models, thus avoiding overfitting. To enhance the generalization of the models, we expanded the SS dataset for training and testing by randomly flipping, rotating, scaling, and mirroring 10 times.

The proposed models are trained for 30 epochs with Adam [48] optimization at the initial learning rate of 0.001, and moments are regulated by two decaying factors $\beta_{1}$ and $\beta_{2}$. Authors suggest that these parameters be initialized to standard values $\beta_{1}=0.9$ and $\beta_{2}=0.999$. These values were applied to our experiments. The learning rate of each parameter group decay by gamma once the number of epoch reaches one of the milestones. The multiplicative factor of the learning rate decay gamma is 0.1. The index of last_epoch=−1 and the list of epoch indices milestones = [15,25]. When last_epoch=−1, the last epoch training learning rate was set as the initial learning rate. When the training epoch is equal to the value in the list of milestones, the learning rate will be updated once by the following formula

(9) $$l_{r}=l_{initial}\times {\left(gamma\right)}^{bisect\_right(milestones,epoch)}$$

where l_r represents the current learning rate, l_initial represents the initial learning rate. bisect_right is the function that returns epoch position in the list of the milestone, and all models are trained one by one.

#### 3.5.3. Experimental Models

We put the DVH module in ➀ and ➁, as shown in Figure 1. We also tried to insert v9h9 and DVH into one model at the position of ➀ and ➁. We did not use any block in the position of this model if the position had a sign of × in Table 1. All design models were trained independently and shared the same training parameters and experimental settings. To compare the effect of the DVH module and the traditional dilated convolution with normal kernel shapes on the final segmentation results, we also designed a basic comparison model, named UNet-D. It uses a 3 × 3 dilated convolution kernel in position ➁; the dilated rate is 2. The other parts of its neural network structure are the same as in Figure 1.

### 3.6. Metrics

The same metrics as in the binary segmentation tasks in references [6,7,8,9,38,46,49,50,51] are applied here. Pixel Accuracy (PA), Mean Pixel Accuracy (MPA), Recall, Precision, and F1 score are used to evaluate our models’ segmentation performance, and can be calculated by the following formula

(10) $$\begin{array}{cc} \; & Accuracy=\frac{TP+TN}{TP+TN+FP+FN}\\ \; & Precision=\frac{TP}{TP+FP}\\ \; & Recall=\frac{TP}{TP+FN}\\ \; & MPA=\frac{\sum_{i=1}^{C}P_{i}}{c},\left(i=0,1,,,c\right)\\ \; & F1=2\cdot \frac{Precision\cdot Recall}{Precision+Recall} \end{array}$$

The semantic segmentation network is a classification network for every pixel: TP is true positive, FP represents false positive, FN stands for false negative, TN means true negative, $P_{i}$ equals to the *i*th class pixel accuracy and *c* is the whole class. Precision and recall are employed as two metrics for a more fair and reasonable comparison, which is defined as F1. For example, in the lane segmentation task, TP means the number of lane pixels that are correctly predicted as lanes. TN means the number of the background pixels which are correctly predicted as the background. FP represents the number of background pixels that are wrongly predicted as lanes. FN denotes the number of lane pixels that are wrongly predicted as the background.

Among these metrics, we pay much more attention to the pixel accuracy and F1 score. These values aim to verify whether or not the VH-stage is useful enough to extract vertical and horizontal linear features. All metrics of different models are obtained under the best threshold.

### 3.7. Results

In this study, we compared experimental results with the state-of-art methods that have been proposed to solve the segmentation results on the SS dataset and the TuSimple dataset. Next, we compared our methods with other segmentation models under the same training conditions, and some of them were proposed recently. Through these two experiments, the influence of the loss function and experimental parameter settings on the comparison results can be excluded, and the effectiveness of our method can be strictly verified.

#### 3.7.1. Comparison with State-of-the-Art Methods

We implemented segmentation networks on the TuSimple dataset and Massachusetts Roads Dataset. The SS dataset was released last year, and there are few experimental results on this dataset. The multi-category segmentation results on the SS dataset in [1] and the segmentation accuracy of slot marking was 74.31%. Our method aiming at binary-category segmentation, and the accuracy of slot markers segmentation is 86.40%.

Many researchers proposed all kinds of methods to improve the lane segmentation accuracy based on the TuSimple lane dataset. Together with the accuracy, false-positive and false-negative lane boundaries are evaluated on the TuSimple lane test dataset. Given that our detected lane boundaries are over 1 pixel in width, we average the x coordinates of the detected pixels for a given row to obtain a single value. The false-positive and false-negative lane boundaries can be calculated as follows

(11) $$\begin{array}{c} FP_{lane}=\frac{F_{pred}}{N_{pred}}\\ FN_{lane}=\frac{M_{pred}}{N_{gt}} \end{array}$$

where $F_{pred}$ represents the wrongly predicted lanes, $N_{pred}$ denotes the number of predicted lanes, $M_{pred}$ means the number of missed ground-truth lanes and $N_{gt}$ indicates the number of all ground-truth lanes.

We calculated the average of inference time on 256 × 160 images for all open source lane segmentation models on an NVIDIA RTX 2080 graphics card. As shown in Table 2, the method proposed by Zou et al. [46] obtains the lowest $FN_{lane}$, but the average inference time of this method is longer. The LaneNet [3] has a faster inference speed and a lower $FN_{lane}$. Our method does not have the lowest average inference time, while it has made progress in metric values of accuracy (97.53%) and $FP_{lane}$. Compared with other open-source models, the average inference time shows that our method has achieved a compromise between accuracy and inference speed. The proposed model can be modernized to reduce computational cost. For example, depthwise separable convolutions can be used to replace the traditional convolutions, and lightweight networks can be used to replace the Unet as the backbone.

#### 3.7.2. An Comparison with Different Models

We compared the segmentation results of the proposed models with FCN, Unet, HFCN, VH-HFCN on the SS dataset, and the TuSimple lane dataset. FCN was a VGG-based segmentation model, and the fully connected layers were transformed into convolution layers in the basic VGG-16 model. FCN-based models, such as HFCN and VH-HFCN, use pre-training VGG-16 as the basic feature extraction layers. We use the same loss function and experimental settings to train those models to verify if our designed models are effective under the same conditions. The SS dataset contains four classes: slot marking, vehicle, free space, and other objects. The model we designed aims for linear feature extraction, and thus the metric that relates to slot marking will reflect our model performance directly. We list the pixel accuracy of slot marking and the metrics (MPA, F1) that reflect the performance of the model shown in Table 3.

As can be seen from Table 3, the UnetDVH-Linear (v1) performs well for slot marking segmentation on the SS dataset. The recall of slot marking segmentation is 88.19%. This result is 2% more than the VH-HFCN network that uses the v9h9 block in the VH-stage. Other metrics that reflect the model on the whole classes (background and slot marking) are MPA and F1 score. Our model gets higher results, and the MPA and F1 of the UnetDVH-Linear (v1) model on the SS dataset is 94.16% and 86.14%, respectively.

According to Table 4, we can conclude that the UnetDVH-Linear (v1) model performs well for road lane segmentation on the TuSimple lane dataset. The precision of lane segmentation is 66.31%. This result is 1% more than the VH-HFCN. Compared with other models, our model obtained the best results on the TuSimple lane dataset with the metric value of MPA (82.87%), Recall (78.33%), and F1 (71.82%).

The experimental verification on the SS test dataset and the TuSimple lane test dataset shows that the DVH block we designed and the network model structure we proposed in this research are more efficient than the traditional linear feature extraction method, such as VH_HFCN [2].

#### 3.7.3. Experiments with Different VH-Stage

We compared the placement of DVH block and v9h9 block in positions ➀ and ➁ to observe the impact of VH-stage on the semantic segmentation network. This is shown in Figure 1. According to Table 5, the UnetDVH-Linear (v1) put the DVH block behind the encoder perform best on metrics value of recall (88.19%) and MPA (94.16%) for slot marking segmentation. In addition, we find that putting the DVH block behind the encoder is more efficient than putting the DVH block inside of the encoder. Compared with UnetDVH-Linear (v1), there is no noticeable effect on the final segmentation result when we put the DVH block inside or behind the encoder (v2, v3). It is clear that the position of the v9h9 (v6) block in and behind the encoder significantly influences slot marking segmentation and has a higher metric value of F1 score (86.69%) and precision (85.58%).

Compared with Unet, FCN, HFCN, and VH-HFCN, it is clear that the F1 score of the slot marking and lane segmentation of the models we proposed has increased 1.5%. On the SS dataset and the TuSimple dataset, the UNet-D model that using traditional dilated convolution with regular kernel shapes improves the effect of linear feature target segmentation, but the model using dilated convolution with horizontal and vertical kernels has a better segmentation effect for linear targets.

According to Table 6, we can find out that the UnetDVH-Linear (v4) we designed to put the v9h9 block behind the encoder performs the best F1 score (71.95%) for road lane segmentation on the TuSimple lane test dataset. We find that the DVH block and the v9h9 block behind the encoder work well with linear feature extraction (v1, v4). This finding is consistent with the experimental results of our first step in Table 3 and Table 4. Regardless of the v9h9 block or the DVH block, VH-stage will be more effective when we put it behind the encoder layers. On the TuSimple lane dataset, all of the models we designed have achieved an improvement in accuracy for lane segmentation, this further verifies the importance of VH-Stage for different linear feature segmentation.

#### 3.7.4. An Comparison with the v9h9 and the DVH Block

The results of the second step in Table 4 and Table 5 indicated that putting the v9h9 block behind the coding layer in UnetDVH-Linear (v4) obtains a better result in the TuSimple lane dataset. To further compare the difference between the v9h9 and the DVH block on the linear feature segmentation, we carried out the exchange of v9h9 and DVH module location experiments in models of UnetDVH-Linear (v7) and UnetDVH-Linear (v8).

The experimental results in Table 7 and Table 8 indicated that the DVH block is more stable than the v9h9 block in encoder layers. The UnetDVH-Linear (v8) obtains the best metric of recall for slot marking and lane segmentation on the SS dataset (88.03%) and the TuSimple dataset (78.08%). These results are nearly 2% higher than other state-of-the-art methods, such as HFCN and VH-HFCN. The comparative experiments in Table 7 and Table 8 shown that when mixing the DVH and v9h9 blocks, placing the DVH block in the encoder layers and placing v9h9 behind the encoder layers can obtain better linear feature extraction results.

#### 3.7.5. Experiments on the Massachusetts Roads Dataset

The SS and TuSimple datasets are collected in street scenes. To verify the segmentation effect of the model on linear features in other scenes, we conducted road segmentation experiments in the aerial images and compared them with other methods. As shown in Table 9, without data augmentation, our method achieved the best accuracy (95.3%), recall (77.60%), precision (77.24%), and F1 (77.42%) for road extraction in the aerial image on Massachusetts Roads Dataset. As shown in Table 10, when using the same loss function and training method, the model we designed improves the precision and F1 of the road segmentation with 1%. On the Massachusetts Roads Dataset, compared with the benchmark models (Unet, HFCN), the UNet-D model that uses dilated convolution with a regular kernel shape does not improve the accuracy of road segmentation. Its precision decreased (57.61%), and the recall increased (72.12%), but the F1 score (64.05%) is not improved.

## 4. Visualization of Results and Discussion

### 4.1. Feature Maps Visualization

**Figure 4:**
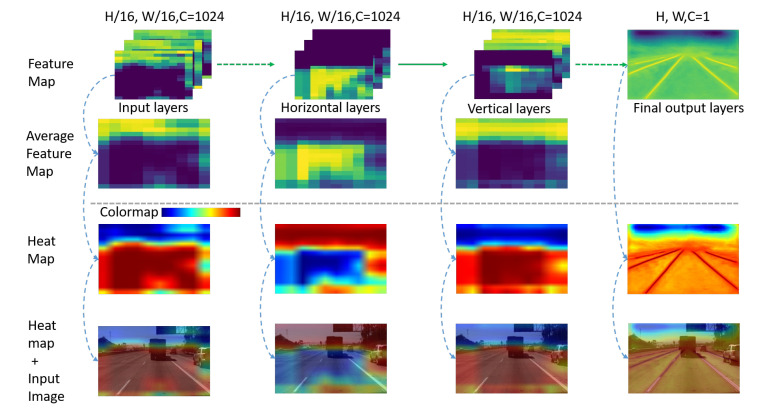
Feature map visualization examples. The first row is the feature map associated with the input and output layers of horizontal and vertical convolutions. For comparison with the final feature map of the output layer, we also show the feature map of the last convolutional layer in the first row. *H* and *W* represent the height and width of the input image, respectively. *C* represents the channel of the convolution layer. In the second row, we use all the feature maps of each convolutional layer to generate an average feature map so that the average value of all channels can be observed. Row 3: The heat map which converts from the average feature map. Row 4: The images generated by merging the heat maps with the input image. In the colormap bar, the color from left to right represents the value from small to large in the average feature map.

To observe and explain the impact of the horizontal and vertical convolutions we designed on linear feature extraction, we visualized the input and output feature maps of horizontal and vertical convolutions on the TuSimple lane test dataset. We also visualized the final layer of the convolutional feature map to compare it with the output feature map of the network. Feature maps of each convolution layer will be resized to the same size as the input image, and then these feature maps will be used to generate an average feature map. To be able to more intuitively view the distribution of the values of the average feature map, we use the average feature map to generate a heat map. Finally, the heat map merged with the original input image with a weight of 0.4. Figure 4 shows the feature maps, heat maps, and final fusion images.

The values in the feature map will be mapped to different colors in the heat map. In the heat map, the redder the color, the greater the value of the corresponding position in the feature map. In this way, how the designed convolution affects linear feature extraction can be observed indirectly from the heat maps.

As shown in Figure 4, the distribution of the input feature map values are almost the same as the feature value after the horizontal and vertical convolutions, but there are also slight differences. Behind the horizontal and vertical convolution kernels, the distribution of the larger values is more concentrated on the road area. The distribution of larger feature map values in the edge of the road area will reduce. This is similar to the heat map of our final output convolutional layer, with larger distribution values in the middle of the road area in feature maps.

**Figure 5:**
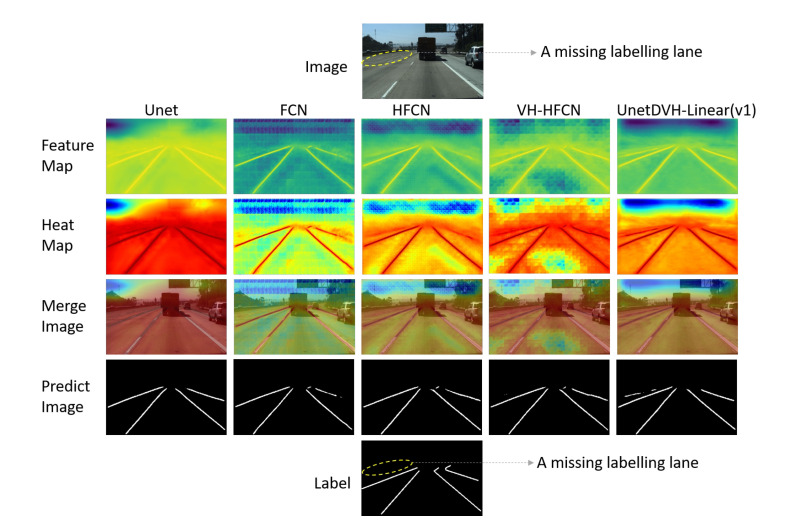
Feature map visualization example of the output layer with different models. There is a missing labeling lane in the label image.

We visualized the output layers of different models on the TuSimple lane test dataset to compare feature maps with different models. As shown in Figure 5, the first row is the input image. From the second row to the sixth row are feature maps, heat maps, merge images, predict images, and labels. The false positive prediction result of the UnetDVH-Linear in Figure 5 is a missing labeling lane. The results demonstrate that our model can capture more details in challenging segmented scenes.

### 4.2. Segmentation Results

**Figure 6:**
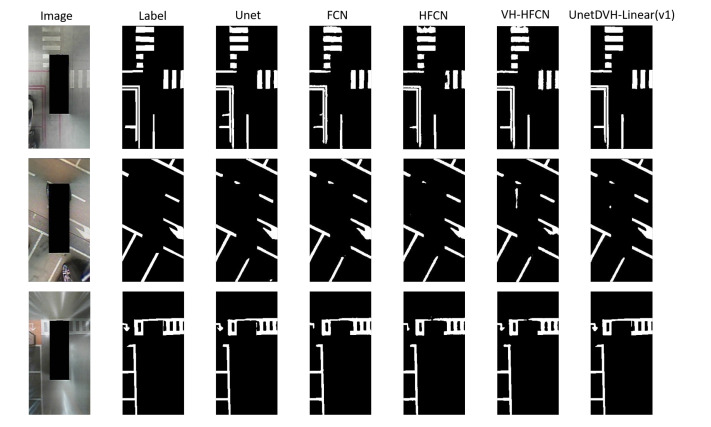
Example of segmentation results on the SS dataset. The first column is input images, and the second column is segmentation masks of the images. Every row represents the segmentation results for the original images of each row with different models. The third column to the sixth column is comparative experimental models. The last column is the model that performs best in our designed model.

**Figure 7:**
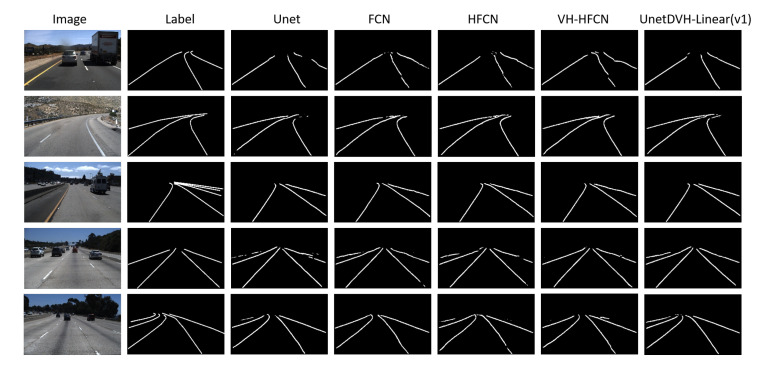
Example of segmentation results on the TuSimple lane dataset. The first column is the input images. The second column is the segmentation masks of the images. The third column to the sixth column is comparative experimental models. The last column is the model that performs best in our designed models.

Our test results on the SS dataset and the TuSimple lane dataset are shown in Figure 6 and Figure 7. On the SS dataset, there is no noticeable difference in the overall segmentation effect. However, the models we designed can capture thin lines, and maintain the smooth edges of linear features. In the second row of Figure 6, we find that the designed model extracts the linear features with missing labeling.

On the TuSimple lane dataset, the experimental results of our designed model and other models are more obvious in Figure 7. The results indicated that the designed model performs better in maintaining smooth and continuous in the lanes segmentation, and the first row can observe these results. Compared to other models, the model we designed can obtain complete segmentation information. In row 4, a lane on the left side of the original label image missed its segmentation annotation label, but our model still obtains this lane segmentation result. All experiment models have lost a segmentation road lane in row 3, but from the example of row 4 and row 5, we can see that the model we designed retains the most complete segmentation information of all lanes, while other comparative models either lose or do not have complete segmentation.

**Figure 8:**
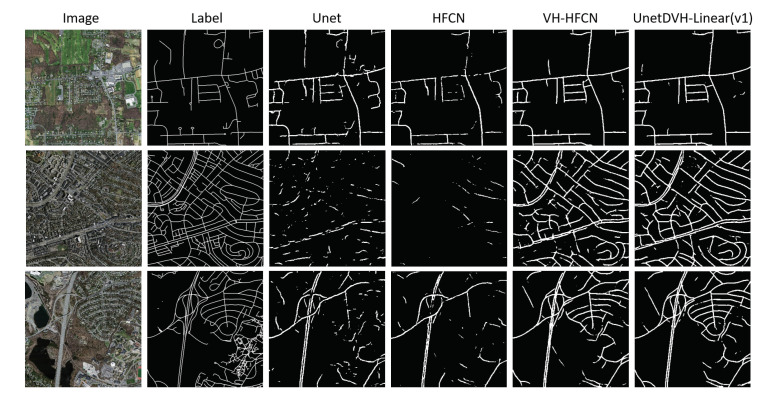
Example of segmentation results on the Massachusetts Road Dataset. The first column is the input images. The second column is the segmentation masks of the images. The third to sixth columns is the segmentation results of different models. The last column is the model that performs best in our designed models.

The segmentation results in the aerial image on the Massachusetts Roads Dataset are shown in Figure 8. In the high-resolution aerial image, our models still perform well.

The intuitive results of these experiments on three different datasets show that the model we designed improved the completeness and continuity of linear feature segmentation. Thus, we obtain higher pixel accuracy of linear feature segmentation when compared to other models.

### 4.3. Discussion

This paper aims to enhance linear feature extraction by adding priori knowledge to neutral networks. We selected parking slots, lanes, and road as experimental objects to observe the effectiveness of the designed models. In a series of comparative experiments, we first compared the effect of the designed model with state-of-the-art models for linear feature extraction. The results in Table 2 suggest that the UnetDVHLinear (v1) have the best accuracy for slot marking and lane segmentation on different datasets. Analyzing the structure of the UnetDVH-Linear (v1) network, we can conclude that this result is related to HF layers and the DVH block. The HFCN and VH-HFCN models have HF layers, and the VH-HFCN model has a VH-stage too. Compared with these models, the neural networks that we designed to add the DVH block for linear feature extraction can be verified. The designed networks take the advantages of the Unet, HF layers, and the VH-stage, and thus can achieve better performance.

Secondly, we changed the position of the VH-stage to compare the effect of the DVH block with the v9h9 block for linear feature extraction. The experimental results in Table 5 and Table 6 show that placing the VH-stage behind the encoder layers (v1,v4) will obtain better results because even if we place the VH-stage in the encoder layers (v2,v5) with a branch, the VH-stage will still destroy feature information learned in the encoder. Thus, the position of the VH-stage will have a big effect on linear extraction.

Finally, we tried to mix the v9h9 block and the DVH block in a different position. When we put the DVH block in the encoder layer and put the v9h9 behind the encoder in a model of UnetDVH-Linear (v8), it obtains excellent results both on the SS dataset and the TuSimple dataset. Moreover, in these group experiments, we find that the DVH block will cause less damage to feature information learned in encoder layers compared with the v9h9 block in encoder layer. From all experimental results, we can figure out that the DVH block is more stable than the v9h9 block in linear feature extraction on different datasets, the v9h9 block in encoder only performs well in the position of ➁ for slot marking segmentation, and the DVH block works well both in the position of ➀ and ➁. These results also relate to the type of the test dataset; all slot markings in the SS dataset are solid lines, but the lanes in the TuSimple dataset have both solid and dashed lines.

The inference speed of our designed model is not the fastest. This is related to the backbone network we used, and the Unet is not a commonly used lightweight network. In future research work, the proposed DVH block can be used in the lightweight semantic segmentation networks. Although the models we designed have achieved relatively favorable results on linear feature extraction, our models have not improved significantly for the metric of precision.

## 5. Conclusions

This paper explored a new method to introduce the dilated convolutions with horizontal and vertical kernels in VH-stage. We constructed an HF layer behind the decoder layer of Unet, and tried to apply different types of horizontal and vertical convolution kernels in the VH-stage at different positions on neutral networks. All the experimental results show that our best models improved the accuracy of the slot marking, roads, and lanes in public datasets. From the experimental results, we found that the performance of the DVH block we designed is more stable than the v9h9 block for the linear feature extraction on a different datasets. Regardless of the types of horizontal and vertical convolution in VH-stage, the VH-stage module putting in the encoder will destroy the information that is learned from encoder layers, even if we put it in the encoder with a single branch. Compared with the v9h9 block, the DVH block we designed caused less damage to encoder learning information, so we can mix the DVH block and the v9h9 block for linear feature extraction; the DVH block can be put in the encoder layer, while v9h9 must be put after the encoder. The v9h9 block will cause irreparable damage to information that is learned by the lower layers. Our model can be used for lane segmentation, parking slots segmentation, and road segmentation. The method proposed in this paper will be extremely beneficial to lane classification and 3D lane detection tasks for the straight lines and dashed lines, and lane segmentation is the first and most important step of these tasks.

Since the training loss functions for binary classification are different from multi-category segmentation, the designed models are trained on the datasets containing background and linear objects (slot markers, lanes, roads) to verify the segmentation performance of the model for linear features. In the next step, we will explore how DVH blocks will affect multi-class segmentation, and how priori knowledge will help us to design spatial convolution kernels for geometric shape segmentation (such as circles and rectangles). We found that many lanes were missing in the TuSimple lane dataset, which will affect the training results of the designed models. In future work, we will relabel such data and publish it to all researchers.

## Figures and Tables

**Table 1:** Through putting different blocks at 1 and 2 in Figure 1, we can explore how the VH-stage may be applied in the networks by designed different models.

Models	➀	➁
VH-HFCN [2]	×	v9h9
Unet-D (ours)	×	Dilated convolution [kernel size is (3,3), dilted factor is (2,2), stride=1]
UnetDVH-Linear (v1) (ours)	×	DVH
UnetDVH-Linear (v2) (ours)	DVH	×
UnetDVH-Linear (v3) (ours)	DVH	DVH
UnetDVH-Linear (v4) (ours)	×	v9h9
UnetDVH-Linear (v5) (ours)	v9h9	×
UnetDVH-Linear (v6) (ours)	v9h9	v9h9
UnetDVH-Linear (v7) (ours)	v9h9	DVH
UnetDVH-Linear (v8) (ours)	DVH	v9h9

**Table 2:** TuSimple lane marking challenge leaderboard (test set) as of 14 March 2018 [46].

Rank	Methods	Name on Board	Using Extra Data	Accurcay (%)	${\mathrm{FP}}_{\mathrm{lane}}$	${\mathrm{FN}}_{\mathrm{lane}}$	Average Inference Times (ms)
2	Zou et al. [49]	UNet ConvLSTM	True	97.3	0.0416	0.0186	47.61
3	Unpublished	leonardoli	-	96.9	0.0442	0.0197	-
4	Pan et al. [50]	SCNN	True	96.5	0.0617	0.0180	18.63
5	Hsu et al. [51]	N/A	False	96.5	0.0851	0.0269	-
6	Ghafoorian et al. [46]	TomTom EL-GAN	False	96.4	0.0412	0.0336	-
7	Neven et al. [3]	LaneNet	False	96.4	0.0780	0.0244	5.04
8	Unpublished	li	-	96.1	0.2033	0.0387	-
9	Pizzati et al. [38].	Cascade-LD	False	95.24	0.1197	0.0620	5.71
1	Ours (v1)	N/A	False	97.53	0.0279	0.0339	12.51

**Table 3:** Quantitative results of Parking slots segmentation performance on SS dataset (%).

Methods	Precision	Recall	MPA	F1
Unet [22]	84.29	86.37	92.29	85.31
FCN [23]	85.03	86.02	92.33	85.52
HFCN [36]	83.07	86.29	91.76	84.65
VH-HFCN [2]	81.79	86.94	91.07	84.29
UnetDVH-Linear (v1)	84.68	88.19	94.16	86.40

**Table 4:** Quantitative results of segmentation performance on the TuSimple lane dataset (%).

Methods	Precision	Recall	MPA	F1
Unet [22]	65.41	77.73	82.40	71.03
FCN [23]	65.88	75.38	82.61	70.31
HFCN [36]	65.08	77.09	82.23	70.58
VH-HFCN [2]	65.57	76.52	82.47	70.61
UnetDVH-Linear (v1)	66.31	78.33	82.87	71.82

**Table 5:** Compared with different position of VH-stage for DVH block and v9h9 block on SS dataset (%).

Methods	Precision	Recall	MPA	F1
Unet [22]	84.29	86.37	92.29	85.31
FCN [23]	85.03	86.02	92.33	85.52
HFCN [36]	83.07	86.29	91.76	84.65
VH-HFCN [2]	81.79	86.94	91.07	84.29
Unet-D	85.38	87.62	92.84	86.48
UnetDVH-Linear (v1)	84.68	88.19	94.16	86.40
UnetDVH-Linear (v2)	84.81	87.43	92.57	86.10
UnetDVH-Linear (v3)	85.42	87.32	92.87	86.36
UnetDVH-Linear (v4)	85.22	88.12	92.79	86.64
UnetDVH-Linear (v5)	84.90	87.78	92.62	86.31
UnetDVH-Linear (v6)	85.80	87.59	93.07	86.69

**Table 6:** Compared with different position of VH-stage for DVH block and v9h9 block on the TuSimple lane test dataset (%).

Methods	Precision	Recall	MPA	F1
Unet [22]	65.41	77.73	82.40	71.03
FCN [23]	65.88	75.38	82.61	70.31
HFCN [36]	65.08	77.09	82.23	70.58
VH-HFCN [2]	65.57	76.52	82.47	70.61
Unet-D	66.07	77.65	82.75	71.39
UnetDVH-Linear (v1)	66.31	78.33	82.87	71.82
UnetDVH-Linear (v2)	66.79	76.41	83.08	71.27
UnetDVH-Linear (v3)	66.90	76.71	83.14	71.48
UnetDVH-Linear (v4)	66.40	78.51	82.91	71.95
UnetDVH-Linear (v5)	66.14	77.70	82.78	71.46
UnetDVH-Linear (v6)	66.45	77.20	82.92	71.42

**Table 7:** Compared the DVH and v9h9 block on the SS test dataset (%).

Methods	Precision	Recall	MPA	F1
Unet [22]	84.29	86.37	92.29	83.79
FCN [23]	85.03	86.02	92.33	85.36
HFCN [36]	83.07	86.29	91.76	86.04
VH-HFCN [2]	81.79	86.94	91.07	84.12
UnetDVH-Linear (v3)	85.42	87.32	92.87	86.36
UnetDVH-Linear (v6)	85.80	87.59	93.07	86.69
UnetDVH-Linear (v7)	85.08	87.75	92.71	86.40
UnetDVH-Linear (v8)	84.83	88.03	92.59	86.40

**Table 8:** Compared with different position of VH-stage for DVH block and v9h9 block on the TuSimple lane test dataset (%).

Methods	Precision	Recall	MPA	F1
Unet [22]	65.41	77.73	82.40	71.03
FCN [23]	65.88	75.38	82.61	70.31
HFCN [36]	65.08	77.09	82.23	70.58
VH-HFCN [2]	65.57	76.52	82.47	70.61
UnetDVH-Linear (v3)	66.90	76.00	83.14	71.48
UnetDVH-Linear (v6)	66.45	77.20	82.92	71.42
UnetDVH-Linear (v7)	66.61	76.70	83.00	71.30
UnetDVH-Linear (v8)	66.20	78.08	82.81	71.65

**Table 9:** Performance of road extraction in Massachusetts Roads Dataset by our method and other approaches (%).

Methods	Data Augmentation	Accuracy	Precision	Recall	F1
Jan et al. [7]	False	82.5	40.5	32.2	35.9
Jan et al. [6]	False	89.9	47.1	67.9	55.6
Zhong et al. [8]	False	90.4	43.5	68.6	53.2
Wei et al. [9]	False	92.4	60.6	72.9	66.2
UnetDVH-Linear (v1)	False	95.3	77.24	77.60	77.42

**Table 10:** Performance of road extraction in Massachusetts Roads Dataset by our method and other models with the same training settings (%).

Methods	Data Augmentation	Precision	Recall	F1
Unet [22]	False	65.40	63.91	64.64
HFCN [36]	False	73.00	65.32	68.94
VH-HFCN [2]	False	75.85	77.07	76.45
Unet-D	False	57.61	72.12	64.05
UnetDVH-Linear (v1)	False	77.24	77.60	77.42

## Abbreviations

The following abbreviations are used in this manuscript:

FCN	Fully Convolutional Network
CNN	Comvolutional Netural Network
DVH	Dilated convolution with vertical and horizontal kernels
HOG	Histogram of Oriented Gradient
LSTM	Long Short-Term Memory
MPA	Mean Pixel Accuracy
PA	Pixel Accuracy
TP	True Positive
TN	True Negative
FP	False Positive
FN	False Negative
GAN	Generative Adversarial Network
BCELoss	Binary Cross-Entropy Loss
GPU	Graphics Processing Unit
RAM	Random Access Memory
HF	High Fusion
VH-stage	Vertical and Horizontal stage
log	Logarithmic Function
R	Set of Real Numbers
$\beta_{1}$	A float value or a constant float tensor. The exponential decay rate for the 1st moment estimates.
$\beta_{2}$	A float value or a constant float tensor. The exponential decay rate for the 2nd moment estimates.
gamma	The multiplicative factor of the decay learning rate.
ms	Millisecond
$km^{2}$	Square Kilometers
*r*	Receptive field
σ	ReLU function
$d_{n}$	Dilated factor
*s*	Stride in Convolution Layers
*k*	Kernel size
